# Role of insulin-like growth factor 1 receptor signalling in cancer

**DOI:** 10.1038/sj.bjc.6602627

**Published:** 2005-05-24

**Authors:** O Larsson, A Girnita, L Girnita

**Affiliations:** 1 Department of Oncology and Pathology, CCK R8:04, Karolinska Hospital, S-171 76 Stockholm, Sweden

**Keywords:** IGF-1, IGF-1 receptor, p53, MDM2, cancer

## Abstract

The insulin-like growth factor (IGF-1) signalling is highly implicated in cancer. In this signalling the IGF-1 receptor (IGF-1R) is unquestionable, the predominating single factor. IGF-1R is crucial for tumour transformation and survival of malignant cell, but is only partially involved in normal cell growth. This is in part due to the interactions with oncogenes. Recent findings suggest a close interplay with the p53/MDM2 pathway. Disturbances in components in the p53/MDM2/IGF-1R network may cause IGF-1R upregulation and growth advantage for the cancer cell. Targeting of IGF-1R is more and more seen as a promising option for future cancer therapy. Single chain antibodies and small molecules with selective effects on IGF-1R dependent malignant growth are of particular interest. Forthcoming clinical trials are welcome and will indeed be the only way to evaluate the impact of IGF-1R targeting in human cancer.

Mounting of evidence provided during the 10 last years implicates a crucial role of insulin-like growth factor 1 (IGF-1) signalling in development and progression of cancer. The most important single component in this signalling, involving the ligands IGF-1 and IGF-2, several binding proteins, proteases as well as three receptors, is the IGF-1 receptor (IGF-1R). In this review we will summarise relevant studies on the role of IGF-1R in cancer with focus on: (1) the unique role of IGF-1R signalling in malignant cells; (2) the interactions between IGF-1R and tumour suppressor genes and proto-oncogenes; as well as (3) current attempts in targeting the IGF-1R as a potential option in cancer therapy.

## UNIQUE ROLE OF IGF-1R IN CANCER

The IGF-1R is a phylogenetically conserved receptor TK and belongs to the insulin receptor family, involving also the insulin receptor (IR), hybrid receptors and the IGF-2R/mannose 6-phosphate receptor. The function of the hybrid receptor is still not well understood. The IGF-2R/6-mannose receptor is a monomeric receptor without TK activities. Both IGF-1R and IR are preformed dimeric TK receptors made up by two extracellular *α*-subunits and two *β*-subunits involving a small extracellular domain, an intramembraneous one as well as an intracellular domain (Adams *et al*, 2000). The latter includes the juxtamembraneous domain, the TK domain and the C-terminal domain. The IGF-1R and IR are highly homologous, especially in the TK domain in which they share 84% amino-acid identities. However, despite these similarities, the functions between IGF-1R and IR differ considerably. The IGF-1R is mainly involved in regulation of cell proliferation, antiapoptosis, differentiation and cell motility, whereas IR is mostly of impact for control of glucose uptake and metabolism. However, the isoform A (exon 11-splice variant) of IR (IR-A), normally expressed in fetal tissues, promotes cell growth in response to IGF-2 stimulation and has been reported to be abundantly expressed in some IGF-1R deficient leiomyosarcomas (Sciacca *et al*, 2002).

The ligand−receptor interaction results in phosphorylation of tyrosine residues in the IGF-1R TK domain (spanning amino acid 973−1229) of the *β*-subunit. The crystal structure of the inactive and phosphorylated kinase domain of the IGF-1R has provided a molecular model of the IGF-1R catalytic activity (Favelyukis *et al*, 2001). In unstimulated state, the activation loop (a-loop), containing the critical tyrosine (Y) residues 1131, 1135 and 1136, behaves as a pseudosubstrate that blocks the active site. Y1135 (being the first tyrosine to be phosphorylated after stimulation with ligand) in the a-loop is bound in cis position in the active site, thus preventing the substrate access and occluding the ATP binding site as well. After ligand binding, the three tyrosines of the a-loop are transphosphorylated by the dimeric subunit partner. Phosphorylation of Y1135 and Y1131 destabilises the autoinhibitory conformation of the a-loop, whereas phosphorylation of Y1136 stabilises the catalytically optimised conformation of it (Favelyukis *et al*, 2001). These changes of the a-loop conformation allow the substrate and ATP access, further increasing the intrinsic TK activity towards phosphorylation of other tyrosines in the receptor and subsequently of exogenous substrate proteins. In turn, phosphorylation of the adaptor proteins insulin receptor substrate 1−4 (IRS-1−4) and Shc leads to activation of the phosphatidyl inositol-3 kinase (PI3K), the mitogen-activated protein kinase (MAPK) and the 14-3-3 pathways (Baserga, 2000).

In contrast to IR, IGF-1R is ubiquitously expressed in tissues in which it plays a role in tissue growth, mostly via the growth hormone, which liberates IGF-1 to activate the IGF-1R. Although this impact in normal growth, strong evidence has been provided that IGF-1R is not an absolute requirement for normal growth, only partially (Baserga, 1999).

On the other hand, IGF-1R has been shown to be crucial for anchorage independent growth, a property being well established to be unique for malignant cells. Classic experiments performed in Baserga's laboratory have demonstrated that anchorage dependency can be acquired when the number of IGF-1Rs at the cell surface is increased to certain amount (Baserga, 1999). The critical role of IGF-1R in anchorage independent growth has been confirmed in several other laboratories. This property of IGF-1R also implicates the function of this receptor in tumour progression since the degree of anchorage independency reflects the level of malignancy. This means that metastasis has acquired more anchorage independency, and more IGF-1R dependency, compared to the primary tumour.

Several studies, both experimental and clinical, have demonstrated that the IGF-1R is overexpressed compared to normal tissues (Belfiore *et al*, 1999; Xie *et al*, 1999; All-Ericsson *et al*, 2002). Furthermore, epidemiological prospective studies have identified high plasma levels of IGF-1 as a potential risk factor for several malignancies (Hankinson *et al*, 1998). In addition, IGF-2, whose expression normally is strictly controlled by parental imprinting is upregulated and functions as an important stimulant of the IGF-1R in cancer (LeRoith and Roberts, 2003). Thus, upregulation of IGF-1R and its ligands are probably important events for the malignant cell growth.

Another aspect on a unique role of IGF-1R in cancer is based on several recent findings that loss of suppressor oncogenes as well as activation of proto-oncogenes is related to IGF-1R function and activity (Baserga, 1994; Girnita *et al*, 2000; Werner and Le Roith, 2000; Werner *et al*, 2000; Girnita *et al*, 2003; Zhao *et al*, 2004). This area of research deserves a separate attention and is discussed in the next section of this review.

### Interactions between IGF-1R and oncogenes

The IGF-1R gene is constitutively expressed in most cells. The promoter of IGF-1R is CG-rich and lacks TATA and CCAAT elements (LeRoith *et al*, 1995b), but has elements found in housekeeping genes, containing regulatory elements characteristic for highly regulated genes (Werner *et al*, 1991). The IGF-1R promoter exhibits a high basal transcriptional activity, and is under physiological control of nutritional factors (Olchovsky *et al*, 1993), hormonal stimulation (Clarke *et al*, 1997) and the developmental stage (Bondy *et al*, 1992). Its expression is altered in certain diseases, including cancer (LeRoith *et al*, 1995a; Baserga *et al*, 1997).

The IGF-1R promoter is targeted by several oncogenes. Constitutive overexpression of the proto-oncogenes c-myb in Balb/c-3T3 cells increases expression of both IGF-1 and IGF-1R, at least partly through an increased transcriptional activity and in this way abrogates the requirement for IGF-1 in the growing media (Kim *et al*, 1996). The hepatitis B virus X protein is another oncogene known to stimulate IGF-1R promoter activity (Kim *et al*, 1996) that may therewith play an aetiologic role in development of hepatocellular carcinoma. Some of the oncogenes increasing the IGF-1R promoter activity can also affect IGF-1R action by nontranscriptional mechanisms. For instance, transformation of human cells by the src oncogene of the Rous sarcoma virus results in constitutive phosphorylation of the receptor *β*-subunit, but addition of IGF-1 further increases the level of phosphorylation (Werner and Le Roith, 2000).

The IGF-1R gene contains several binding sites for members of *ERG* family of transcription factors. Wilms' tumour 1 gene (*WT1*), a member of this family, is a tumour suppressor gene and its product has been shown to suppress the activity of promoters containing WT1 binding sites. Genes with WT1 targeting promoters include *IGF-1R* and *IGF-2*. WT1 has been shown to bind to the IGF-1R promoter and to suppress activity (Werner *et al*, 1995). Consistently, loss of *WT1* activity in Wilms' tumour and related malignancies may result in transcriptional derepression of the *IGF-1R* gene (Gerald *et al*, 1995). Pathologic fusion of the Ewing gene *EWS* to *WT1* (t(11;22)(p13;q12)(*EWS/WT1*)) has been shown to abrogate the tumour suppressor function of *WT1* and to generate an oncogenic chimeric protein capable of binding and activating the *IGF-1R* promoter (Karnieli *et al*, 1996).

Likewise, the suppressor oncogene p53 is capable of suppressing the activity of the IGF-1R promoter as well as lowering the endogenous levels of IGF-1R mRNA (Werner *et al*, 2000). In addition, the transcription of the IGF-2 gene is similarly reduced by wild-type p53 (Zhang *et al*, 1996). In contrast, tumour-derived, mutant versions of p53 significantly stimulated promoter activity (Werner *et al*, 1996). These data therefore suggest that upregulation of IGF-1R due to loss-of-function of p53 may facilitate selection of a malignant population of cells. However, the role of p53 in regulation of IGF-1R seems to be more complex and probably also involves post-transcriptional mechanisms (Girnita *et al*, 2000; Lee *et al*, 2003). This can be exemplified by malignant melanoma cells, most often harbouring wild-type p53 (Hussein, 2004), which exhibit overexpression of IGF-1R. Upon inhibition of wild-type p53 in these cells, they surprisingly responded with a drastic IGF-1R downregulation and cell death (Girnita *et al*, 2000). Similar results have been obtained in other studies (Lee *et al*, 2003). These observations points to the action of other mechanisms in the p53-dependent control of IGF-1R expression. Such a mechanism could theoretical be mediated by the oncoprotein MDM2, which interacts with p53 but has recently also been found to associate with certain cell surface receptors and regulate their functions (Shenoy *et al*, 2001). MDM2 is well known to strictly control p53. Overexpression of MDM2 results in decreased level and activity of p53 (Kubbutat *et al*, 1997) and provides an alternative to a ‘p53 mutation’ in the sense that it inactivates p53. In this way, MDM2 enables the development of tumours that retain wild-type p53. Just, recently, it was shown that under conditions when p53 was inhibited, MDM2 was redistributed and bound to the IGF-1R (Girnita *et al*, 2003). MDM2 was proven to ubiquitinate the IGF-1R and degraded it in a proteasome-dependent manner (Girnita *et al*, 2003), eventually leading to cell death. This action of MDM2 explains the earlier results that inhibition of wild-type p53 unexpectedly leads to downregulation of the IGF-1R (Girnita *et al*, 2000). These data are in consistent with several other studies reporting apoptotic effects due to overexpressed MDM2 (Vousden and Prives, 2005). However, drastic redistributions of MDM2 from p53 to the IGF-1R can probably only be achieved after experimental modulations, like inhibition of p53 expression by antisense strategies, etc, and do not likely occur in this manner in a physiologic context. On the other hand, an increased distribution of MDM2 to the cell nucleus to interact with p53 may indirectly increase the expression of IGF-1R since lesser cytoplasmic MDM2 will be available to ubiquitinate and degrade the receptor. Unpublished studies in our laboratory have suggested that some amounts of MDM2, by inducing ubiquitination, are important for internalisation and activation of the IGF-1R. However, if the MDM2 levels are becoming too abundant, the IGF-1R is over-ubiquitinated and degraded. These findings are also interesting from an IGF-1R targeting point of view because pharmacological modulations leading to an excess of MDM2 could be a manner to cause a selective IGF-1 inhibition and apoptosis in cancer cells.

Reciprocally, the IGF-1 system has been shown to influence the activity of MDM2. IGF-1 was demonstrated to regulate MDM2 activity by inhibiting the association between p19ARF and MDM2 in a p38 MAPK-dependent manner (Heron-Milhavet and LeRoith, 2002). Thus, when IGF1 was used to rescue the cells from UV-induced DNA damage, the p53 protein was degraded through the MDM2-mediated pathway. Others studies indicate that expression of phosphorylated Akt increases MDM2-mediated ubiquitination of p53 (Mayo and Donner, 2001). The serum-induced increase in p53 ubiquitination was blocked by a PI3K inhibitor, suggesting that phosphorylated Akt enhances the ubiquitination-promoting function of MDM2, determining reduction of the p53 protein.

**Figure 1:**
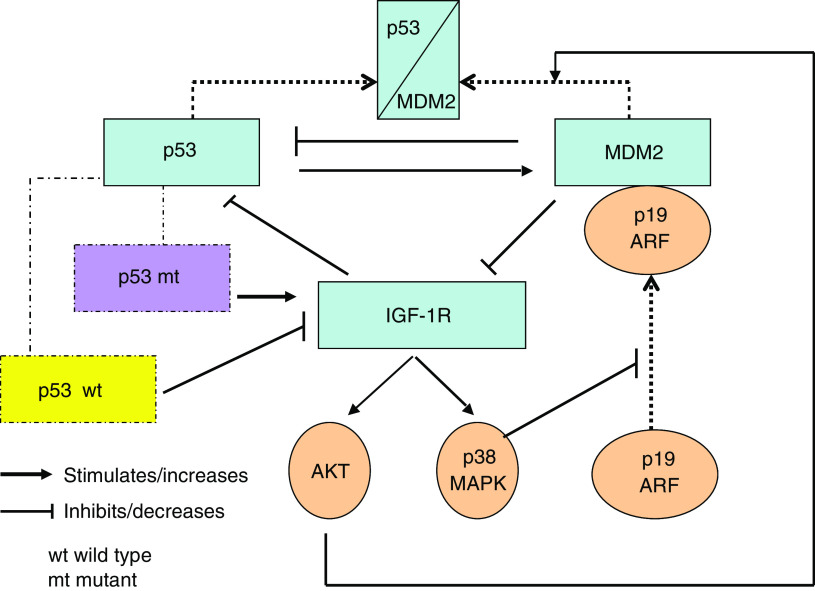
Interplay between p53, MDM2 and IGF-1R. The upper part of scheme shows that MDM2 can decrease p53 synthesis but also associate (indicated by dotted arrows) to it. This causes ubiquitination of p53. On the right it is indicated that p19 ARF can associate (dotted arrow) to MDM2. This prevents MDM2 association to p53.

In conclusion, there seems to exist a p53/MDM2/IGF-1R axis, in which signals are propagated in either direction. Changes leading to increased distribution of MDM2 to the cell nucleus to inactivate p53 may contribute with a growth advantage for the tumour cells by upregulating the IGF-1R. This could be due to a derepressed transcription of the *IGF-1R* gene as well as a decreased ubiquitination and degradation of the receptor. A schematic picture illustrating possible links between p53 and IGF-1R is presented in Figure 1.

## TARGETING IGF-1R IN CANCER

The vast expression of IGF-1R in neoplastic cells and tissues combined with its crucial roles in cancer cell growth is making this tyrosine receptor an attractive target to combat malignant diseases.

Blockade of IGF-1R has been convincingly shown to cause massive apoptosis of tumour cells *in vivo*, to inhibit tumorigenesis and block tumour invasion and metastasis. Overall, strategies leading to downregulation of the receptor, and not only inhibition of its TK activity, have been associated with the strongest antitumour efficacies (Baserga *et al*, 2003). This may be due to that a downregulation of IGF-1R is necessary to produce a complete inhibition of its function.

A variety of approaches aimed at targeting IGF-1R has been utilised to prove the concept, or are being developed for potential anticancer therapies. Targeting of IGF-1R to block its signalling may be obtained by interference with ligand/receptor interactions, receptor synthesis and expression, receptor TK activity, or combinations of these strategies.

Strategies aimed to block the ligand–receptor interaction involve receptor neutralising antibodies (Kalebic *et al*, 1994). Among those most studied is the monoclonal antibody *α*-IR3, which competes with IGF-1 for binding to the receptor and blocks receptor activation (Van Wyk *et al*, 1985). However, *α*IR3 can sometimes act as an IGF1 mimetic and especially in cells overexpressing the IGF-1R (Kato *et al*, 1993). Antibody blockade of IGF-1R has been attempted in breast cancer model systems. However, the large size of the therapeutic molecule restricts its access to tumour cells, particularly in central regions of solid tumours (Russell *et al*, 1992). Smaller fragments are currently being studied as a substitute for whole antibodies in an effort to improve access and uptake. Sachdev *et al* (2003) used a single-chain antibody directed against IGF-1R (IGF-1R scFv-Fc) to examine the effects on IGF-1R signalling. *In vivo* treatment of mice bearing MCF-7 xenograft tumours with scFv-Fc resulted in near complete downregulation of IGF-1R.

Dominant-negative mutated IGF-1R (Dunn *et al*, 1998; Brodt *et al*, 2000) and truncated soluble IGF-1R (D'Ambrosio *et al*, 1996) are two related strategies to block IGF-1R. D'Ambrosio *et al* engineered by a frame-shift mutation a human IGF-1R cDNA that produces 486 amino acids long receptor. This truncated soluble receptor inhibited the autophosphorylation of the endogenous IGF-1R as well as induced extensive apoptosis *in vivo* and inhibited tumorigenesis in syngeneic rats. From a therapeutic point of view, these strategies suffer from the problem how to administrate these molecules to receive an efficient uptake in the tumour cells.

Antisense techniques are another way to inactivate the IGF-1R. Resnicoff *et al* (1994) used antisense RNA to IGF-1R by introducing it into cells by either addition of oligodeoxynucleotides or by transfection with plasmids expressing antisense RNA to IGF-1R RNA. Injection of glioblastoma cells (C6) IGF-1R antisense cells into rats carrying an established wild-type C6 tumour caused complete regression of the tumours. This fact further raises the possibility of practical applications targeting IGF-1R. Moreover, downregulation of IGF-1R, obtained by antisense strategies, has been reported to elicit a host response leading to eradication of surviving malignant cells *in vivo* (Resnicoff *et al*, 1996). Interestingly, in a pilot study, exposure of autologous glioma cells treated *ex vivo* with IGF-1R antisense oligos induced partial tumour regression in some patients with malignant astrocytoma (Andrews *et al*, 2001). This response seems to be immunogenic, involving the MHC I system, but is still not closer characterised.

A direct strategy to interfere with IGF-1R activity is to induce selective inhibition of its TK by developing selective small-molecular inhibitors. The major advantage of this approach is that small molecules have a considerable higher bioavailability compared to antibodies, dominant-negative receptors and antisense oligonucleotides. However, TK inhibitors face the problem that IGF-1R and IR are so similar. Actually many of the hitherto developed IGF-1R TK inhibitors have also caused substantial inhibition of the IR. Such cross reaction would probably cause diabetic reactions in patients and can therefore not be accepted. On the other hand, IR-A dependent tumours would not be affected by a fully selective IGF-1R inhibitor.

Most of the IGF-1R TK inhibitors produced so far have served as competitive ATP inhibitors. Since the region of the TK domain covering the ATP binding site is identical to that of the IR, such cross-inhibitions are not unexpected. However, there is a recent interesting exception. Garcia-Echeverria *et al* (2004) presented a new compound (a pyrrolo[2,3-d] pyrimidine) that although inhibiting the IGF-1R and IR TK equipotently in cell-free systems, exhibited several-fold selectivity for the IGF-1R in a cellular context and reduced the growth of IGF-1R positive fibrosarcomas *in vivo*.

Blum *et al* (2003) presented a new family of bioisostere inhibitors, based on the structure of AG 538, a tyrphostin inhibiting the IGF-1R TK at the substrate level and not at the ATP binding site (Blum *et al*, 2000). These AG 538 bioisosteres possessed similar but weaker biological properties to AG 538 but are more stable and blocked the formation of colonies of prostate and breast cancer cells in soft agar systems (Blum *et al*, 2003).

Recently, we demonstrated that the cyclolignan PPP inhibited phosphorylation of IGF-1R without interfering with insulin receptor activity (Girnita *et al*, 2004), as well as it reduced phosphorylated Akt, caused apoptosis and induced tumour regression in xenografted mice. PPP did not compete with ATP but interfered with phosphorylation in the activation loop of the kinase domain, in which it specifically blocked phosphorylation of the tyrosine (Y) 1136 residue, while sparing the two others (Y1131 and Y1135). Since an IGF-1R construct, in which the tyrosine at position 1136 was replaced by a phenylalanine, also led to a strong inhibition of phosphorylated Akt in transfected cells, it was suggested that this mechanism may be responsible for the apoptotic effect of PPP (Vasilcanu *et al*, 2004). Unpublished studies have also demonstrated that the IGF-1Rs of PPP treated cells are undergoing rapid downregulation. This downregulation may be important for the strong apoptotic effect of this compound. Table 1 summarises different approaches to target the IGF-1R.

Although a huge number of experimental and preclinical investigations have provided encouraging results, clinical trials must be performed and completed to definitely evaluate the usefulness and risks of targeting IGF-1R as an option in cancer treatment of humans.

## Figures and Tables

**Table 1:** Approaches to target IGF-1R

**Site of inactivation**	**Way of inactivation**		**Reference**
Synthesis/expression	Antisense oligodeoxynucleotides		Pietrzkowski *et al* (1993), Resnicoff *et al* (1994), Andrews *et al* (2001)
	Antisense techniques with plasmids		Resnicoff *et al* (1994)
	RNA interference		Gray *et al*, 2003
	Triple helix strategy		Rininsland F *et al* (1997)
			
Expression	Dominant-negative receptors		Dunn *et al* (1998), Reinmuth N *et al* (2002), Scotlandi *et al* (2002)
	Truncated soluble receptors		D'Ambrosio *et al* (1996)
			
Ligand–receptor interaction	Blocking antibodies	*α*IR3	Van Wyk (1985)
	Single chain antibodies	scFv-Fc	Sachdev *et al* (2003)
			
Tyrosine kinase activity/expression	Small molecules	Tyrphostin bioisosteres	Blum *et al* (2003)
		Pyrrolo[2,3-d] pyrimidine derivatives	Garcia-Echeverria *et al* (2004)
		Cyclolignan PPP	Girnita *et al* (2004)
